# Management of Endometrial Cancer: A Comparative Review of Guidelines

**DOI:** 10.3390/cancers16213582

**Published:** 2024-10-24

**Authors:** Stergios Kopatsaris, Ioannis Tsakiridis, Georgios Kapetanios, Fotios Zachomitros, Georgios Michos, Evangelos Papanikolaou, Apostolos Athanasiadis, Themistoklis Dagklis, Ioannis Kalogiannidis

**Affiliations:** Third Department of Obstetrics and Gynaecology, School of Medicine, Faculty of Health Sciences, Aristotle University of Thessaloniki, Konstantinoupoleos 49, 54642 Thessaloniki, Greece; stkopats@auth.gr (S.K.); kapetaniosg@hotmail.gr (G.K.); fzachomitros@auth.gr (F.Z.); gmichos@auth.gr (G.M.); evpapanikolaou@auth.gr (E.P.); apathana@auth.gr (A.A.); dagklis@auth.gr (T.D.); ikalogia@auth.gr (I.K.)

**Keywords:** gynecological cancer, endometrial cancer, guidelines, management, comparison

## Abstract

Endometrial cancer is the most frequently diagnosed gynecological cancer. The aim of this study was to summarize and compare the most recent guidelines regarding its management. A comparative review of guidelines from the European Society of Gynecological Oncology, the European Society for Radiotherapy and Oncology, the European Society of Pathology, the European Society for Medical Oncology, the Cancer Council of Australia, the British Gynecological Cancer Society, the National Comprehensive Cancer Network, and the American College of Obstetricians and Gynecologists was carried out. The comparison of guidelines highlighted common recommendations and key differences regarding the most basic aspects of endometrial cancer management. Certain differences were identified in the options of surgical procedures based on endometrial biopsy and lymph node infiltration. Minor differences were identified in the management of recurrent disease, while the more pronounced differences were found in the follow-up procedure. The development of consistent international protocols in similar resource settings may lead to a more consistent classification, as well as to common lines regarding surgical approaches, risk stratification and the way patients should be monitored following the remission of endometrial cancer.

## Simple Summary

This is a comparative review of guidelines on the management of endometrial cancer. Since, endometrial cancer is a common gynecological pathology, especially in women of postmenopausal age, it is crucial to develop consistent protocols on the management of the disease. In this descriptive review, we managed to compare the recommendations from six influential guidelines on endometrial cancer. This comparison may offer valuable information to the clinicians managing such patients.

## 1. Introduction

Endometrial cancer is the most frequently diagnosed gynecological cancer [1], constituting the fourth most common type of cancer in high-income countries [2]. It mostly affects postmenopausal women; early-onset (i.e., diagnosed before the age of 50) endometrial cancer is relatively uncommon as its incidence is 6.9 per 100,000 women [3]. In two-thirds of the cases, the patients are diagnosed with localized disease which is associated with a 5-year survival rate of 95% [4].

With regard to risk factors, non-genetic characteristics and medical conditions have been associated with endometrial cancer [5]. In particular, hypertension, diabetes, and other diseases associated with elevated estrogen levels, such as late menopause and high body mass index (BMI), are known risk factors for this type of cancer [6]. Smoking and certain patterns of dietary intake have also been associated with a higher risk of endometrial cancer [7,8]. On the contrary, the use of certain contraceptive medications reduces the risk of developing endometrial cancer [9].

There are two main histological types of endometrial cancer, type 1 and type 2 [10]. The staging of the disease is based on several parameters, including tumor grade, myometrial invasion, lymph node involvement, as well as the involvement of the ovaries and the uterus [11,12,13]. One of the most important elements in the diagnosis and treatment of endometrial cancer during the last decade has been the molecular classification of specific carcinomas; this categorization aims at evaluating the disease recurrence risk, and therefore the probability of cancer survival [14].

Several national and international medical societies have developed guidelines to help improve clinical practice, especially optimize therapeutic protocols. The aim of this descriptive review was to summarize and compare major guidelines with regard to the management of endometrial cancer.

## 2. Evidence Acquisition

The most recently published guidelines on the management of endometrial cancer based on the FIGO classification [15] were retrieved and a comparative review was conducted. More specifically, six guidelines were identified from: the National Comprehensive Cancer Network (NCCN 2023) [16], the consensus among the European Society of Gynecological Oncology (ESGO), European Society for Radiotherapy and Oncology (ESTRO) and European Society of Pathology (ESP) (2020) [17], the Cancer Council of Australia (CCA 2020) [18], the European Society for Medical Oncology (ESMO) (2022) [19], the American College of Obstetricians and Gynecologists (ACOG) [20], and the British Gynecological Cancer Society (BGCS 2022) [21].

**Figure 1:**
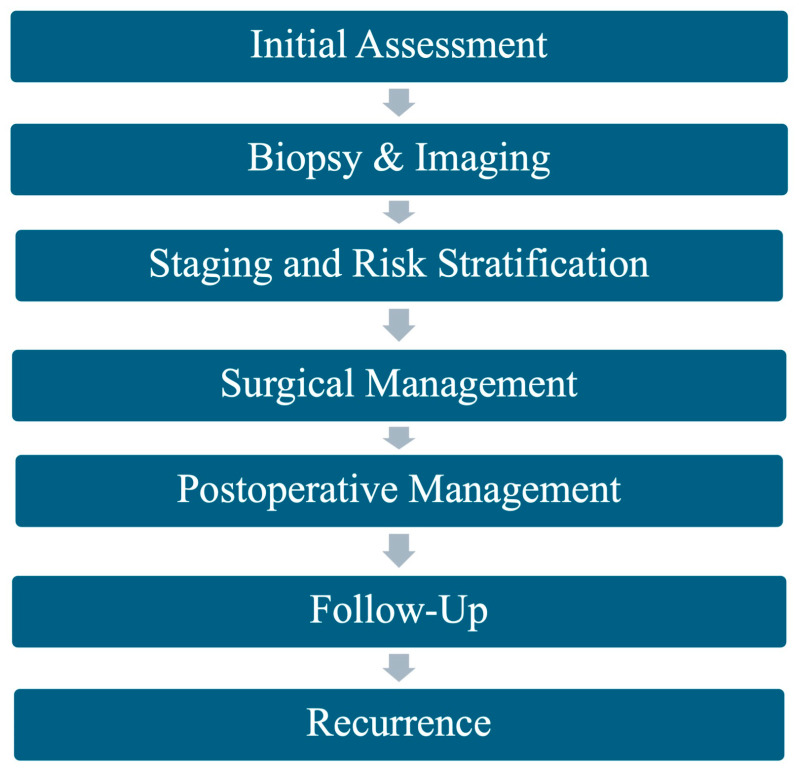
Management of endometrial cancer.

**Figure 2:**
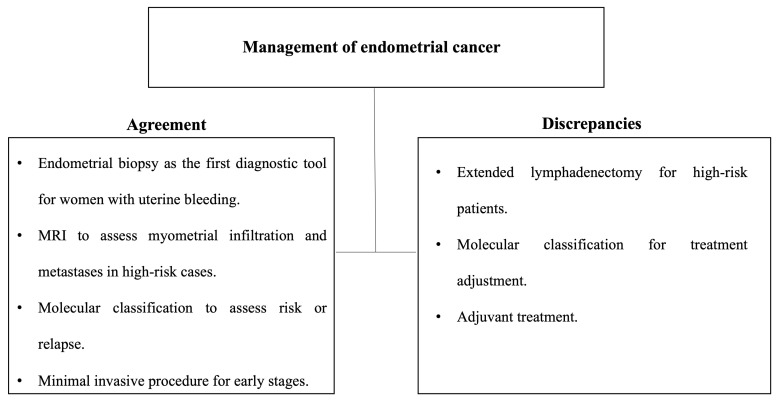
Points of agreement and disagreement among guidelines on the management of endometrial cancer.

An overview of the recommendations (Figure 1 and Figure 2) is presented in Table 1 (preoperative management), Table 2 (surgical management), Table 3 (sentinel lymph node assessment) and Table 4 (adjuvant therapy).

## 3. Diagnosis and Preoperative Management

Preoperative management involves endometrial biopsy and imaging examinations [22]. The majority of endometrial cancer patients experience vaginal bleeding, mostly during the postmenopausal period [23]; even though strategies aiming at early detection have the ability to detect up to 90% of cases, only up to 10% of women with abnormal bleeding will receive an adequate diagnosis [24]. Therefore, it is critical to identify high-risk patients in order to implement the appropriate therapeutic/interventional protocol and increase survival rates [25]. To establish a diagnosis, a histological examination is required. The initial evaluation for diagnosed or suspected endometrial cancer, as recommended by all the reviewed guidelines, includes a detailed medical history, as well as a complete gynecological examination and a transvaginal scan. Moreover, the NCCN, ACOG, CCA and BGCS guidelines propose endometrial biopsy as the first diagnostic tool for those patients presenting with uterine bleeding. The Australian and British guidelines, as well as ACOG suggest that initial biopsy should be attempted using a pipelle; in cases where the results are inconclusive, a dilation and curettage should be performed [18,20,21]. ESMO suggests that both pipelle biopsy and curettage under anesthesia are acceptable first line approaches for histological diagnosis [19]. On the contrary, the ESGO-ESTRO-ESP guideline does not specify the biopsy method [17]. The choice of biopsy assessment is crucial for several reasons; different biopsy methods may have different cancer detection rates [26]. Moreover, choosing the appropriate method affects patient experience and comfort, as some procedures may be more invasive than others [27]. Overall, each approach has advantages and disadvantages.

NCCN, based on studies that evaluated the clinical worth of imaging techniques [28,29], suggests that preoperative management may include a frontal chest radiograph, leaving the option of a pelvic magnetic resonance imaging (MRI) open, in order to determine the tumor’s origin and evaluate the possibility of potential metastases. Additional imaging examinations (computerized tomography—CT and/or positron emission tomography) may be performed to evaluate metastasis based on clinical manifestations or if the chest X-ray reports suspicious findings. In cases of clear cell carcinomas or serous or undifferentiated carcinomas where a thoracic/abdominal/pelvic MRI has been performed, CT should be carried out preoperatively to assess for the presence of ectopic disease [16]. Likewise, the ESGO-ESTRO-ESP and ESMO guidelines, based on relevant studies [30,31], state that MRI is rather convenient, as it is adequately precise for the appraisal of the myometrial invasion’s depth and possible metastases to the lymph nodes. CCA endorses imaging examination in cases where there are clinical signs indicating metastasis or in cases where the histological examination suggests high-risk disease. A CT scan of the abdominal-pelvic region is suggested when clinicians suspect either high-grade carcinomas or metastatic tumors. MRI is suggested for the assessment of cervical involvement and this is considered useful for patients who want to maintain their fertility. ACOG considers MRI as an option for further evaluation when clinicians detect either high-risk histology or signs of metastasis. However, ACOG focuses attention on individualized preoperative management plans based on the histology examination. As for BGCS, MRI is proposed in cases of high-risk patients to assess myometrial invasion and cervical involvement.

Regarding the prognostic factors, all the reviewed guidelines agree on the central role of stratifying endometrial cancer patients into low, intermediate, and high-risk groups for the peritoneal disease, as well as future recurrence and unfavorable prognosis. This method of classification assists decision-making for the possible lymphadenectomy and the application or not of adjuvant treatment such as chemo- or radiotherapy to reduce the risk of recurrence and manage prognosis effectively.

The molecular classification provides in-depth information regarding morphological characteristics and it is supported that it should be incorporated both in the pathological report of the endometrial biopsy, as well as in the postoperative sampling results [32]. Molecular classification is also the procedure that will guide clinical management when chemotherapy is a potential option. In cases where molecular classification is not available, the ESGO-ESTRO-ESP guideline suggests that cancer classification should be based on standard clinical practices. NCCN along with ACOG encourage the use of molecular classification as a tool for complementary assessment of the type of carcinoma. On the other hand, ESGO-ESTRO-ESP, ESMO and BGCS use molecular classification only to characterize the degree of cancer risk.

## 4. Surgical Management

The basis of surgical treatment in patients with endometrial cancer involves total hysterectomy with bilateral salpingo-oophorectomy (TAH/BSO), as well as lymph node evaluation [33]. In premenopausal patients, ovarian preservation may be an option if the disease is of low risk as relevant research suggests this approach may improve survival rates for patients of young age with early-stage disease [34].

The surgical approach may be laparoscopic, robotic, vaginal or abdominal; the typical procedure followed for those with disease confined to the endometrium is minimally invasive surgery, as this ensures a lower rate of postoperative infection, bleeding, and venous thromboembolism and reduces the required hospitalization time, without compromising the outcome of the disease [35]. NCCN, ESGO-ESTRO-ESP, and ESMO recommend minimally invasive methods when the surgical approach is necessary, even in patients with high-risk endometrial cancer. CCA underscores that a laparoscopic approach should be performed when it is considered feasible and safe, as it offers a reduced possibility for post-surgical impediments and limited hospitalization. Similarly, ACOG supports minimally invasive surgery, such as laparoscopy, based on individualized disease characteristics and patient’s health status. BGCS also favors minimally invasive procedures (laparoscopy) and states that the choice of treatment should be based on the intraoperative findings as well as the risk assessment. On the other hand, metastatic uterine, cervical or lymph node tumors are not candidates for minimally invasive techniques; in these patients, the first-line choice of treatment is TAH/BSO with surgical staging.

In advanced-stage cases, ESGO-ESTRO-ESP recommends peritoneal biopsy; ESMO does not support peritoneal sampling for cases of clear cell carcinomas, due to the low probability of metastasis. In stages III and IV of endometrial cancer (including sarcoma), maximal cyto-reduction should only be considered if complete gross removal is feasible, a recommendation that applies to all the reviewed medical societies. Limited evidence shows that this may improve progression-free and overall survival rates in patients with advanced-stage or recurrent endometrial cancer [21]. Peritoneal cytology that results in positive findings is considered a negative prognostic factor for recovery from the disease, although it does not affect staging. NCCN and ESMO support the collection of cytological samples, as these may be useful for clinical decisions.

## 5. Evaluation of the Lymph Nodes

Lymph node assessment is considered crucial in surgical management, as it contributes to important predictive information that may facilitate therapeutic interventions [36]. The standard procedure involves the removal of the lymph nodes (outer and inner iliac), with or without the removal of the paraaortic lymph nodes [37].

Sentinel lymph node biopsy has been introduced as an alternative to lymph node resection because it provides similar predictive accuracy with the latter, while it minimizes the chances of postoperative complications, such as lymphedema of the lower extremities [38]. CCA suggests that lymph node assessment should not be performed for early-stage and low-grade disease and is in favor of this approach only for patients with high-risk or high-grade histology, whose health status does not allow full surgical staging. ESGO-ESTRO-ESP states that mapping of the sentinel lymph nodes can be considered in low- or intermediate-risk patients, while BGCS is in favor of the sentinel lymph node biopsy in clinically early-stage disease and underscores the performance of ultra-staging and immunohistochemistry to augment the detection of possible micro-metastases for high-risk patients. The presence of macro- and micro-metastases (<2 mm) should be considered as metastatic involvement and indicates a worse prognosis [15]. For patients with high-risk tumors, the ESGO-ESTRO-ESP guidelines recommend lymphadenectomy, including pelvic and paraaortic resection, while the sentinel node procedure is an acceptable alternative procedure to the systematic lymphadenectomy. Similarly, NCCN recommends sentinel lymph node biopsy as the preferred practice for lymph node evaluation, for patients with tumors confined to the uterus and only if this is feasible. Notably, ACOG recommends paraaortic lymphadenectomy solely for high-risk diseases with deep invasive lesions, serous or clear carcinomas or carcinosarcomas and in cases where the myometrium invasion is not reported.

## 6. Fertility Preservation

Fertility preservation treatment is suggested and offered only to carefully selected patients with low-grade tumors that wish to maintain their reproductive ability [39]. ACOG, NCCN, ESGO-ESTRO-ESP, ESMO, as well as BGCS, advise patients to refer to specialized clinics and receive detailed advice for treatments that protect fertility. Moreover, while they promote fertility preservation, the ESGO-ESTRO-ESP and ESMO guidelines focus on molecular categorization for a more personalized approach, while NCCN does not. Based on specific scientific data, ESGO-ESTRO-ESP states that this treatment should only be considered by experienced gynecological oncologists using well-defined protocols with detailed patient information and close follow-up and only for patients with atypical hyperplasia/endometrioid intra-epithelial neoplasia or grade 1 endometrioid carcinoma without myometrial invasion [40,41]. CCA follows a slightly different approach, where MRI is considered an additional procedure for evaluating the ability to preserve fertility. If this can be achieved, hormonal therapy may be considered, but only for patients with early-stage and low-grade cases. ACOG and BGCS align with CCA, as they promote hormonal augmentation (progestin) only for patients with grade 1, stage IA endometrial cancer with no myometrial invasion. They also propose close follow-up to ensure patient safety. TAH/BSO is recommended for cases where childbearing is complete, if the treatment is not effective (maintenance of disease after 6 months of progestin—NCCN) or if the disease recurs. The ESGO-ESTRO-ESP guidelines are in favour of hysteroscopic biopsy due to its higher accord with the final clinical judgement compared to curettage, while NCCN does not make a specific recommendation between the two methods. ESGO-ESTRO-ESP guidelines, for these patients, emphasize the need for intravaginal ultrasound or MRI evaluation to rule out myometrial infiltration or cervical invasion. Treatment consists of continuous intake of progestin and may include megestrol, medroxyprogesterone or the placement of an intrauterine device with levonorgestrel, while for optimum results the combination with oral progesterone intake is suggested [42]. ESGO-ESTRO-ESP and ESMO guidelines recommend that hysteroscopic biopsy be preceded by progestogens since existing data show that when this procedure is followed, a higher rate of complete remission of the disease is achieved [43]. NCCN recommends follow-up by either biopsy or curettage every 3–6 months. On the contrary, ESGO-ESTRO-ESP and ESMO guidelines recommend closer follow-up with hysteroscopic biopsy at 3 and 6 months; if the disease is treatment-resistant post 6 months, standard surgical procedure is advised.

## 7. Adjuvant Therapy

Adjuvant therapy plays an important role in the management of endometrial cancer, complementing primary treatment. As already mentioned, surgery is the primary procedure for tumor removal, but, adjuvant treatment i.e., chemotherapy, radiotherapy, and hormonal treatment may sufficiently assist in eliminating those cancer cells that have not been removed completely [44,45]. Chemotherapy may be effective in advanced diseases, while radiation therapy can shrink the tumor before or after surgery [46]. In addition, hormonal therapy may be effective in hormone-dependent cancers [47]. In general, adjuvant therapy can improve treatment outcomes, reduce the risk of cancer recurrence and improve the quality of life of endometrial cancer patients [48].

Adjuvant therapy depends largely on the prognostic risk group that the patient is classified; a discrepancy is noted between some of the reviewed guidelines. The ESGO-ESTRO-ESP and ESMO guidelines suggest different interventions based on the five risk categories (low, intermediate, intermediate/high, high-risk, and advanced disease). More specifically, the ESGO-ESTRO-ESP guidelines, based on randomized trials, do not recommend adjuvant therapy for patients with low-risk disease [49,50,51]. Similarly, CCA and ACOG state that adjuvant treatment should be based on each patient’s clinical profile and suggest radio- or chemotherapy-only for women with high-risk diseases. BGCS does not deviate from the individualized approach of adjuvant treatment for high-risk patients, but additionally, it suggests hormonal therapy for those of low risk, especially for women who wish to preserve their fertility. NCCN, on the other hand, categorizes patients into those with uterine-restricted disease, those with metastatic disease and those with relapsing-metastatic disease. Furthermore, ESGO-ESTRO-ESP and ESMO emphasize the use of molecular categorization to tailor therapy; this allows for a more personalized approach, based on the genetic differences of tumors and their response to different types of treatments. In contrast, NCCN does not take molecular differences into account; this may lead to less personalized treatments and possibly a suboptimal treatment response. Thus, the difference between the guidelines is essentially the approach to personalizing therapy based on the molecular profile of the tumor.

## 8. Conclusions

The comparison of the above-mentioned guidelines highlights significant similarities and differences, in three key areas: the use of molecular classification, surgical staging, and treatment approach. First, in relation to molecular classification, ESGO-ESTRO-ESP and ESMO emphasize its importance in decision-making regarding the therapeutic strategy, revising adjuvant therapy based on the molecular profile. This approach has proven critical in classifying patients and reducing unnecessary procedures. In contrast, NCCN and ACOG are more restrained in their use of molecular classification, following more traditional criteria for deciding treatment. Secondly, with respect to surgical staging, there is disagreement regarding the performance of lymphadenectomy. NCCN and ACOG recommend a more selective approach, based on clinical risk factors, while ESGO-ESTRO-ESP and ESMO encourage more extensive lymphadenectomy if molecular data support it. This differentiation is reflected in the approach to risk management and complication avoidance. Lastly, the application of adjuvant therapy differs, with ESGO-ESTRO-ESP and ESMO recommending tailored treatments based on molecular profiling, while NCCN and ACOG maintains a more conservative approach, based on traditional staging methods. Taken together, these differences demonstrate the evolution of medical practice towards a more individualized approach, particularly in Europe, while more traditional methods still maintain a strong presence in the US.

Different guidelines often bring different perspectives, reflecting the latest research findings and clinical experiences. This diversity ensures that clinicians should be well informed about various aspects of endometrial cancer management, ranging from risk assessment and staging to treatment options and postoperative care. In addition, the combination of guidelines facilitates an individualized approach to patient management. By intersecting various guidelines, especially in settings with no official national guidance, clinicians may tailor their decisions to each patient’s unique individual characteristics, such as age, comorbidities, and tumor histology; this approach could contribute to the most appropriate patient care, tailored to their needs.

## Figures and Tables

**Table 1:** Summary of recommendations on the preoperative management of endometrial cancer.

Association	NCCN	ACOG	CCA	BGCS	ESMO	ESGO-ESTROESP
**Initial evaluation**	Detailed medical history, gynecological examination	Detailed medical history, gynecological examination	Detailed medical history, gynecological examination	Detailed medical history, gynecological examination	Detailed medical history, gynecological examination	Detailed medical history, gynecological examination
**Histology**	Endometrial biopsy	Endometrial biopsy	Endometrial biopsy	Endometrial biopsy	Endometrial biopsy	Not specified
**Imaging**	Frontal chest radiograph, optional pelvic MRI; CT for clear cell, serous, or undifferentiated carcinomas; additional CT or PET scan based on clinical signs	MRI for high-risk histology or signs of metastasis; individualized plans based on histology	Imaging for clinical signs of metastasis or high-risk disease; CT for high-grade or metastatic tumors; MRI for cervical involvement	MRI for high-risk patients to assess myometrial invasion and cervical involvement	MRI for myometrial invasion and lymph node metastases	MRI for myometrial invasion and lymph node metastases
**Prognostic factors**	Stratify into low, intermediate and high-risk groups	Stratify into low, intermediate and high-risk groups	Stratify into low, intermediate and high-risk groups	Stratify into low, intermediate and high-risk groups	Stratify into low, intermediate and high-risk groups	Stratify into low, intermediate and high-risk groups
**Molecular classification**	Encourages use	Encourages use	Not specified	Use only to characterize the degree of cancer risk	Use only to characterize the degree of cancer risk	Use only to characterize the degree of cancer risk

CT: computerized tomography, MRI: magnetic resonance imaging, PET: positron emission tomograph.

**Table 2:** Overview of the surgical management of endometrial cancer.

Degree of Risk	NCCN	ESGO-ESTRO-ESP, ESMO	ACOG	CCA	BGCS
**Low/Intermediate Risk**	**IA and IB** Total hysterectomy with bilateral salpingo-oophorectomy, surgical staging is recommended.	**IA** Minimally invasive approach. Total hysterectomy with bilateral salpingo-oophorectomy. SNLB may be an option. **IB** Minimally invasive approach. Total hysterectomy with bilateral salpingo-oophorectomy. SNLB may be an option Minimally invasive approach. Total hysterectomy with bilateral salpingo-oophorectomy. SNLB may be an option.	**IA and IB** Total hysterectomy and bilateral salpingo-oophorectomy.	**IA and IB** Total hysterectomy and bilateral salpingo-ophorectomy.	**IA and IB** Minimally invasive surgery should be embraced as the standard surgical approach.
**Intermediate High/High Risk**	**II** Cervical biopsy or magnetic resonance imaging of the pelvis. If the result is negative, total hysterectomy with bilateral salpingo-oophorectomy. If the result is positive, total hysterectomy with bilateral salpingo-oophorectomy, with surgical staging **III and IV** Systemic therapy, external radiotherapy and brachytherapy	**IA** Minimally invasive approach. Total hysterectomy with bilateral salpingo-oophorectomy. SNLB may be an option. **IB** Minimally invasive approach. Total hysterectomy with bilateral salpingo-oophorectomy. SLNB or systemic lymphadenectomy **II** Total hysterectomy with bilateral salpingo-oophorectomy. SLNB or systemic lymphadenectomy **III** Any intraperitoneal dispersion of a tumor, including tumor rupture or tissue dissection, should be avoided. If vaginal extraction is at risk of uterine rupture, other measures should be taken (e.g., mini laparotomy, use of endobag). Tumors with metastases outside the uterus and cervix (excluding lymph node metastases) are contraindications for minimally invasive, surgical practices. **IV** Surgical staging is an option for patients who were previously incompletely staged with high-intermediate-risk/high-risk disease, since the outcome may have implications for the adjuvant treatment strategy.	**IA and IB** Minimally invasive approach. Total hysterectomy with bilateral salpingo-oophorectomy. SNLB may be an option. **II** Total hysterectomy, bilateral salpingo-oophorectomy, and consideration of radical hysterectomy **III and IV** Total hysterectomy and bilateral salpingo-ophorectomy.	**IA, IB and II** Minimally invasive approach. Total hysterectomy with bilateral salpingo-oophorectomy. SNLB may be an option.	**IA and IB** Total hysterectomy with bilateral salpingo-oophorectomy. In cases involving cervical stromal invasion, a radical hysterectomy may also be performed. **II** Total hysterectomy, bilateral salpingo-oophorectomy, the extent of lymphadenectomy may be more conservative. A radical hysterectomy may be recommended. **III and IV** Total hysterectomy with bilateral salpingo-oophorectomy, often combined with cytoreductive surgery to remove as much tumor burden as possible.

**Abbreviations:** SNLB: Sentinel Lymph Node Biopsy.

**Table 3:** Recommendations on sentinel lymph node assessment.

Degree of Risk	ESGO-ESTRO-ESP, ESMO	ACOG	CCA	BGCS	NSSN
**Low**	SLN biopsy may be considered for staging purposes in patients with low-risk disease. It can be omitted in cases without infiltration of the myometrium. Systemic lymphadenectomy is not recommended in this group.	SLN biopsy is not recommended for low-risk disease.	SLN biopsy is not recommended for low-risk disease	SLN mapping may be considered in select cases but not routinely recommended for low-risk disease.	SLN is suitable for patients at low risk for metastases and/or those who may not tolerate lymphadenectomy. SLN is suitable for patients at intermediate risk for and/or those who may not tolerate lymphadenectomy. SLN should always be performed before a hysterectomy, except when the uterus needs to be removed. If SLN fails, pelvic lymphadenectomy should be performed and any suspicious or enlarged lymph nodes should be removed.
**Intermediate**	SLN biopsy may be considered for staging purposes in patients with intermediate-risk disease. It can be omitted in cases without infiltration of the myometrium. Systemic lymphadenectomy is not recommended in this group. Pelvic systemic lymphadenectomy should be performed in intermediate-high risk patients if no SLN is detected on either side of the pelvis. Pathological hyperstagation of the SLNs is recommended.	Consider lymph node assessment for intermediate-risk endometrial cancer, particularly if there are other high-risk features present.	Lymph node assessment may be considered for intermediate-risk endometrial cancer, depending on tumor size, histological grade of the tumor, depth of myometrial invasion, presence of lymphovascular invasion, age and overall health of the patient, presence of other comorbidities.	SLN mapping or selective lymphadenectomy may be considered for intermediate-risk disease.	
**High**	Surgical staging of lymph nodes should be performed in patients with high-intermediate/high-risk disease. SLNB is an acceptable alternative procedure. If pelvic lymph node involvement is detected intraoperatively, further pelvic lymph node dissection should be performed. Removal of enlarged lymph nodes and paraaortic staging may be considered.	Lymph node assessment recommended for high-risk endometrial cancer, including systemic pelvic and para-aortic lymphadenectomy.	Lymph node assessment, including pelvic and para-aortic lymphadenectomy, recommended for high-risk endometrial cancer.	Systemic lymphadenectomy is recommended for high-risk diseases.	

SLN: Systemic Lymph Node.

**Table 4:** Summary of recommendations on adjuvant therapy for the management of endometrial cancer.

Degree of Risk	ESGO-ESTRO-ESP, ESMO	NCCN	CCA	ACOG	BGCS
**Low risk**	Adjuvant therapy is not recommended	No adjuvant therapy for stages IA or vaginal brachytherapy if lymphatic infiltration is present and patient age > 60 years or atrial brachytherapy and external radiotherapy in cases of stage IA with lymphatic infiltration	Adjuvant therapy is not recommended	Adjuvant therapy is not recommended	Adjuvant therapy is not recommended
**Intermediate risk**	Vaginal brachytherapy or external radiotherapy (radiation)				Radiation only
**Intermediate-high risk**	Radiotherapy and chemotherapy for lymphovascular space invasion or only vaginal brachytherapy in cases of low lymphatic infiltration		Radiotherapy and chemotherapy in cases of high-grade	Radiotherapy and chemotherapy in cases of high-grade	External radiotherapy and chemotherapy
**High risk**	External radiotherapy and chemotherapy for lymphovascular space invasion, endocervical invasion or stage IIIB-IIIC cancer	External radiotherapy and chemotherapy			
**Advanced disease**	Prior surgery with tumor removal, if macroscopic resection is feasible with acceptable survival rates and potential for improvement of the patient’s quality of life				

## Data Availability

All the data used for this article are publicly available.
